# New Insights on the Scalping Phenomenon of Volatile Sulphur Compounds on Micro-Agglomerated Wine Closures

**DOI:** 10.3390/molecules28135094

**Published:** 2023-06-29

**Authors:** Rémi De La Burgade, Valérie Nolleau, Teddy Godet, Nicolas Galy, Dimitri Tixador, Christophe Loisel, Nicolas Sommerer, Aurélie Roland

**Affiliations:** 1 SPO, Univ Montpellier, INRAE, Institut Agro, 34060 Montpellier, France; remi.de-la-burgade@supagro.fr (R.D.L.B.);; 2 DIAM Bouchage, 3 Rue des Salines, 66400 Céret, France

**Keywords:** volatile sulphur compounds, wine closure, scalping, sorption

## Abstract

Flavour scalping in wine is a well-known phenomenon that is defined as the sorption of flavour compounds on wine closures. While the impact of closure type was the object of several studies, no research has addressed the impact of wine closure permeability on flavour scalping. For that purpose, the adsorption of volatile sulphur compounds (VSCs) on four micro-agglomerated wine cork closures was investigated by soaking them in model and Shiraz wines for 7 days. From a kinetic point of view, most of the VSCs were quickly scalped after 1 h of soaking, and this effect increased after 6 h until reaching a plateau. Most importantly, no significant impact of the closure on the kinetics and adsorption rates of the VSCs was found. As to the quantitative aspects, VSC sorption on closures accounted for 1% to 5% of the initial VSCs present in the wines only, meaning that the impact was negligible under oenological conditions.

## 1. Introduction

The evolution of wine aroma during bottle ageing has been of great interest to guarantee quality over time. Wine is a complex matrix that involves redox reactions often catalysed by oxygen. During bottle ageing, it was demonstrated that wine closures played a role into the chemical reactions by allowing a specific amount of oxygen to interact with wine compounds. Therefore, the choice of closure could be a determinant of wine quality during bottle storage. Cork closures were traditionally used for bottling, but new types of closures emerged over the years, such as synthetic closures, screwcaps, and technological cork closures [1]. Among the latter, micro-agglomerated wine cork closures are produced by binding cork particles with different binders such as polyurethane, melamine, and rubber [2].

While the role of wine closures on the amount of oxygen exchanged is well-known [3], closures, as well as a wide range of other packages, could play a role in the scalping of flavour compounds [4]. Flavour scalping has been described as the sorption of aroma compounds by the packaging material, which could result in losses in flavour intensity. Many parameters can impact the scalping effect, such as the nature of the material and the flavour properties (polarity, molecular size) but also the storage conditions [4]. Other factors such as temperature and humidity were reported to impact the flavour scalping of (R)-limonene on orange juice packaging [5].

The behaviour of volatiles on polymeric packaging is well studied, whereas only few publications focused on flavour scalping in wine closures. Several flavour chemical families have been investigated in wine, such as esters, benzenoids, methoxypyrazines, and volatile phenolic compounds [6,7,8,9]. Indeed, esters such as ethyl hexanoate, ethyl octanoate, and ethyl decanoate could be scalped by wine closures [6]. The effect was greater with synthetic closures (50% to 70% of ethyl decanoate adsorbed on the closure after 2 years of storage) than for technological and natural cork closures (from 20% to 30% sorption). However, no case of scalping was observed for screw cap closures in the same study. For 2-phenylethyl acetate, higher levels were found in wines bottled with natural corks compared to synthetic closures, suggesting a higher scalping effect for synthetic closures due to the polyolefinic material used for its production [10].

3-alkyl-2-methoxypyrazines, another flavour family, are also subjected to adsorption at the closure’s surface [7]. These compounds, such as 3-isobutyl-2-methoxypyrazine (IBMP), 3-*sec*-butyl-2-methoxypyrazine (SBMP), and 3-isopropyl-2-methoxypyrazine (IPMP) were perceived as green and vegetative notes in wine [11,12]. This study, performed on Riesling and Cabernet Franc wines, showed that scalping had the greatest impact on IBMP [7], but all molecules had a higher concentration scalped in bottles closed with synthetic closures compared to natural cork and screwcap closures. The same conclusion was reached on a Chardonnay wine, in which various cork products were soaked for 140 h [13]. Indeed, synthetic cork closures most affected wine concentrations in IPMP, IBMP, and SBMP (decreases of 77%, 70%, and 89%, respectively), followed by agglomerate and natural cork closures. SBMP was the most affected by scalping in this article.

Other works suggested that cork could sorb volatile phenolic compounds [8,9]. The investigation of seven phenolic compounds (guaiacol, 4-methylguaiacol, 4-ethylguaiacol, 4-propylguaiacol, 4-vinylguaiacol, 4-ethylphenol, and 4-(2-propenyl)guaiacol) showed that all of them could be adsorbed on a cork’s surface, with a higher sorption coefficient for the most hydrophobic molecules (from 125 mg/kg for guaiacol to 306 mg/kg for 4-(2-propenyl)guaiacol). The same study suggested that volatile phenols were trapped via weak interactions at the cork’s surface. Moreover, the second study demonstrated the role of suberin, the major component of cork, as the component that had a higher sorption rate with volatile phenols compared to cork. This study, performed on two spoiled wines, showed that a large amount of the initial concentrations of 4-ethylguaiacol and 4-ethylphenol (49.2% and 44.6% for wine A and 66.8% and 71.2% for wine B, respectively) was sorbed by suberin. The adsorption of chloroanisoles by corks has been studied for a long time [14]. Indeed, during bottle ageing, natural cork closures can adsorb 2,4,6-trichloroanisole, 2,3,4,6-tetrachloroanisole, pentachloroanisole, 2,4-dichloroanisole, and 2,6-dichloroanisole from wine in an extremely slow way. These results were relatively identical with coated and uncoated corks.

1,1,6-trimethyl-1,2-dihydronaphtalene (TDN) was observed to follow the same behaviour as volatile phenols: after 2 years of storage of a white wine, there was a higher scalping effect from a synthetic closure (96% and 98% of adsorbed TDN) compared to natural and technical cork closures (50% and 70% of adsorbed TDN, respectively) [6]. In Riesling wines originating from diverse regions around the world, more than half of the TDN contained in the wine was adsorbed by cork and synthetic closures, whereas no TDN loss was observed for bottles closed with screwcaps [15]. The scalping effect from a German Pinot Blanc white wine was also studied [16]. In this work, a TDN solution was added into a wine that was bottled with several types of closures and then stored for 3 to 6 months. For the micro-agglomerated cork closures, the times and temperatures of the storage had significant effects on the scalping of TDN: a higher TDN concentration was scalped after 6 months of storage (75%) compared to 3 months (60%), whereas a higher temperature (27 °C) decreased the amount of scalped TDN (50%), contrasting with a lower temperature (14 °C) that scalped a higher amount (60%). No effect of bottle position (horizontal or vertical) was observed.

Finally, the scalping of volatile sulphur compounds (VSCs) has also been investigated. While excess exposure to oxygen could lead to the wine developing oxidative flavours (rotting apple, green, and earthy notes), a lack of oxygen in the wine could induce reduced off-flavours, often described as rotten egg, cooked cabbage, and asparagus like [17]. The VSCs mainly responsible for these deviations were hydrogen sulphide (H_2_S), methanethiol (MeSH), and DMS (dimethyl sulphide) [18]. The sorption of H_2_S and DMS at the surface of different closures has also been examined [19]. Different types of closures were soaked in a model wine, to which H_2_S and DMS had been added, and the solutions were analysed after 7 and 25 days. After this time, both VSCs showed scalping effects, where both H_2_S and DMS concentrations were mainly affected by synthetic and natural cork closures compared to screwcaps.

As seen above, the type of closure exhibited the same kind of behaviour towards all aroma families, with synthetic closures being the most prone to flavour scalping, followed by natural and micro-agglomerated cork closures, whereas screwcaps showed no scalping phenomenon. Non-polar compounds were found more likely to be sorbed on closures due to their interactions with the closure synthetic materials. Many experiments focused on the differences of the scalping effect between different closure types, but no article was found to study the role of the permeability of a same type of closure. As the “reduction off-flavour” is due to an excess of VSC under anoxic conditions, it could be interesting to investigate the scalping effect as a technological solution to mitigate undesired notes. Therefore, the aim of this study was to investigate the scalping phenomenon of seven VSCs on four different micro-agglomerated wine cork closures from kinetic and quantitative points of view.

## 2. Results

The aim of this study was to understand the evolution of seven VSCs (ethanethiol, dimethyl sulfide, diethyl sulfide, *S*-methylthioacetate, *S*-ethylthioacetate, dimethyl disulfide, and diethyl disulfide) towards the scalping phenomenon of micro-agglomerated wine closures. As these molecules were shown to be responsible for reduced-off flavours during bottle ageing, we, therefore, investigated the role of flavour scalping in counterbalancing these off-flavours.

### 2.1. Development and Optimization of a Sample Preparation for VSC Desorption and Subsequent Analysis by GC-MS/MS

At the beginning of the experiment, the optimal time of desorption was determined: closures from Shiraz wine bottles were desorbed into a model wine to check the amount of VSCs scalped at the closure surface and to conclude on the optimal duration of desorption. The durations tested were 1 h, 2 h, 3 h, 6 h, and 18 h (Figure S1).

For each VSC, the amount desorbed per hour in the model wine was higher at 1 h, 2 h, and 3 h and remained stable after 6 h and 18 h. Therefore, the duration of desorption used for artefact checking was 3 h.

### 2.2. Artefact Checking from New Closures

New wine closures were analysed to check the absence of analytical artefacts. After 3 h of desorption in a model wine, 10 mL samples were analysed, and no VSCs were detected by GC-MS/MS. Consequently, new closures used in this study were free of VSCs (see Section 4.5.2).

### 2.3. Model Wine Study

To clarify the results, the concentrations were expressed as the sum of VSCs (µmol/L) in all figures. The details of the data were displayed in Table S1.

In the model wine, four types of wine closures were tested, as well as a blank that contained no closure to distinguish chemical reactions from sorption reactions. A total of seven VSCs were analysed in model and Shiraz wines at the beginning of the experiment and after 1 h, 6 h, 3 days, and 7 days (see Section 4.5.3). For each sample, the wine contained in each sample was analysed, and the given concentration corresponded to the concentration of the remaining VSCs in the liquid ($C_{R}$) (Table S1).

Over time, there were losses of VSC in the samples (mainly chemical reactions or physical adsorptions), and this concentration corresponded to: $C_{L}=C_{I}-C_{R}$ in the blank samples, with $C_{I}$ being the initial concentration of VSC at t0.

The concentration attributed to the scalping phenomenon was calculated as follows: $C_{S}=C_{I}-C_{R}-C_{L}$ for the samples containing closures.

#### 2.3.1. Evolution of the Kinetic Behaviour of VSCs

##### Total Amount of VSCs

**Figure 1:**
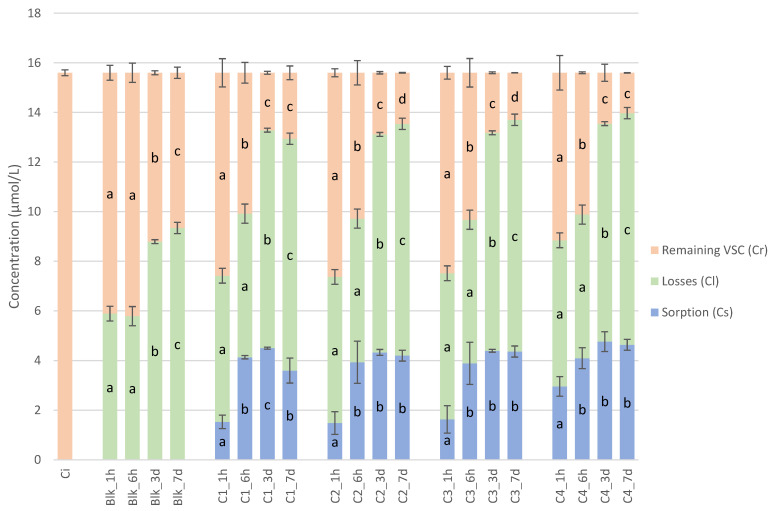
Evolution of total VSCs in model wine and extrapolations (losses were calculated in blank samples and then reported for each closure sample; sorptions were calculated based on the losses in blank samples and the remaining VSCs in closure samples, compared to the initial concentration), depending on the stirring time and the type of closure (the letters a, b, c, and d represent the significant differences at α = 0.05 between stirring times for each closure and the blanks).

The concentrations of the sum of VSC depending on time and the type of closure are presented in Figure 1.

At t0, the total concentration of VSCs in all samples was 15.6 µmol/L. After 1 h of stirring, the concentration of VSCs in blank samples decreased significantly (9.71 µmol/L). In the blank samples, the concentration remained significantly the same after 6 h (9.81 µmol/L) and then continued to decrease significantly (*p*-value = 0.05) at 3 days (6.81 nµmol/L) and 7 days (6.26 µmol/L). Losses were observed after 1 h of stirring, and $C_{L}$ increased significantly after 3 days and even more after 7 days.

All samples with closures showed a scalping phenomenon after 1 h until 7 days. $C_{S}$ increased significantly (*p*-value = 0.05) for all closures from 1 h to 6 h of stirring, and then the concentration remained stable for all closures.

##### Evolution of the Scalping Kinetics for Each VSC

Two types of behaviours were observed: four VSCs increased their scalped concentrations with time (EtSH, SMTA, DMDS, ETA), whereas the other three (DMS, DES, DEDS) first increased and then decreased over time.

For EtSH, there was a scalping effect after 1 h of stirring and no significant evolution after 6 h, 3 days, and 7 days. The SMTA concentration sorbed at the surface of the closures was effective after 1 h and remained significantly constant for all times of analysis except for C1 and C4 that were significantly higher after 3 and 7 days for C1 and only after 7 days for C4 compared to 1 h. All closures scalped DMDS and ETA after 1 h until 7 days, with no significant evolution over time.

For DMS, no scalping effect was observed for C1, C2, and C3 after 1 h; thereafter, all closures demonstrated levels of DMS sorbed at their surfaces after 6 h. For DMS and DES, all closures exhibited bell-shaped curves, with an increase up to 3 days and 6 h, respectively, before a decrease until 7 days. Last, DEDS had a significant higher concentration adsorbed on the four closures at 1 h compared to 3 and 7 days of stirring.

#### 2.3.2. Comparison of the Scalping Effect between Closures

##### Total Amount of VSC

**Figure 2:**
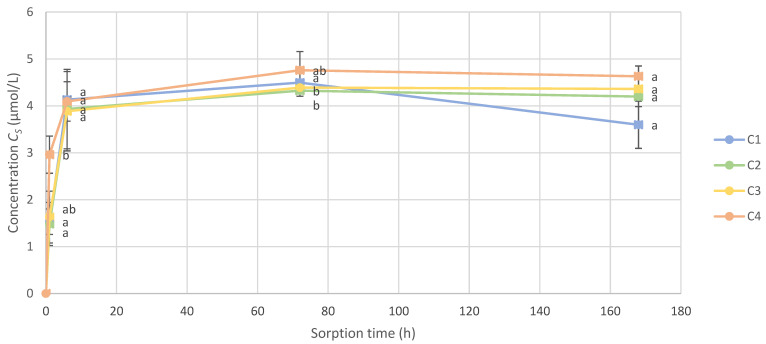
Kinetics of the total amount of VSCs scalped on wine closures ($C_{S}$) in model wine (the letters a and b represent the significant differences at α = 0.05 between closures at each time of measure).

The scalping effect kinetics were monitored and compared among the four types of closures. At each analysis time, C1, C2, C3, and C4 were compared via an ANOVA statistical analysis using a Student’s *t*-test (Figure 2).

In the model wine, at 1 h of stirring, C4 showed a scalped concentration significantly higher compared to C1 and C2. Other closures showed no significant differences between them after 1 h. After 6 h, there were no significant differences between closures, considering the total amount of VSCs. At 3 days of stirring, C1 had a significant higher concentration scalped compared to C2 and C3, whereas all other closures had no differences between them. Finally, at the end of stirring, there were no significant differences between all closures.

##### Comparison of the Scalping Kinetics between Closures for Each VSC

At each time of analysis, each VSC concentration scalped at the surface of the closures were compared, but no significant differences between C1, C2, C3, and C4 were observed for any VSC at any time.

### 2.4. Red Wine Study

#### 2.4.1. Evolution of the Kinetic Behaviour of VSCs

##### Total Amount of VSC

**Figure 3:**
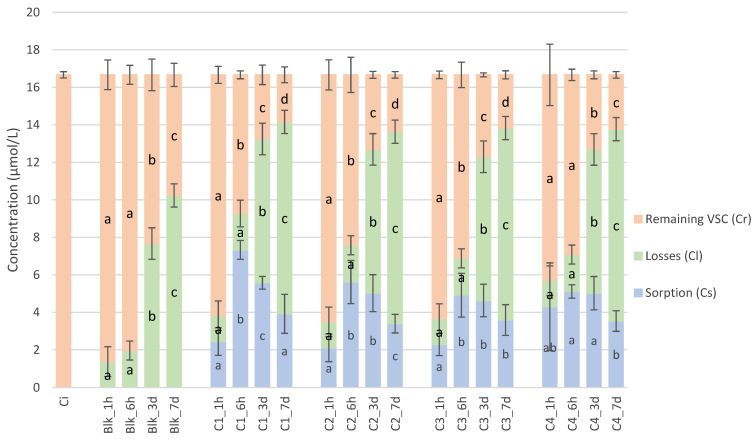
Evolution of total VSCs in red wine and extrapolations (losses were calculated in blank samples and then reported for each closure sample; sorptions were calculated based on the losses in blank samples and the remaining VSCs in closure samples, compared to the initial concentration), depending on the stirring time and the type of closure (the letters a, b, c, and d represent the significant differences at α = 0.05 between stirring times for each closure and the Shiraz wine blanks).

The kinetics of the total amount of VSCs for each closure are displayed in Figure 3.

The calculations performed on model wine ($C_{S}$, $C_{L}$, $C_{I}$, $C_{R}$) were also used in Shiraz wine. All $C_{R}$ values for each VSC are displayed in Table S1.

The initial concentration of the total amount of VSC was 16.7 µmol/L. In the Shiraz wine blank samples, the concentration in the model wine decreased significantly after 1 h of stirring and remained stable at 6 h, with the concentration decreasing significantly after 3 days and even more after 7 days.

Generally, all closure samples showed the same behaviour, with bell-shaped curves described for $C_{S}$ where there was a significant increase between 1 h and 6 h of stirring, then a plateau up to 3 days and, finally, a significant decrease in the concentration scalped after 7 days of soaking.

##### Evolution of the Scalping Kinetics for Each VSC

In red wine, DMS, DMDS, and ETA were found to be scalped at every time of analysis, but no significant evolution was observed (Table S1). For the other compounds, we observed interesting kinetics of scalping with two different behaviours (Table S1).

EtSH, DES, and DEDS all displayed decreasing scalping amounts over time. After 1 h of stirring, all closures had adsorbed EtSH at their surface. For the C1 samples, there was a significant increase at 6 h, and then the concentration decreased after 3 and 7 days to remain significantly equivalent to the 1 h value. No significant variations over time were found on the C2 samples. C3 showed an increase after 6 h compared to the sample at 1 h, whereas no significant further variation was detected. As for the C4 closure, the concentration decreased between 6 h and 7 days of stirring. In the red wine, DES was the only VSC that was adsorbed up until 3 days, and then there were no scalping after 7 days of stirring. The tendencies of the kinetics were generally the same, with bell-shaped curves. C1 and C2 had their scalped concentrations increased between 1 h and 6 h before decreasing significantly at the third day of analysis. The concentrations adsorbed on the C3 and C4 closures were constant between 1 h and 6 h and then decreased significantly over 3 days. The analysis of DEDS showed a similar behaviour of all closure samples, where the concentration decreased significantly between 6 h and 3 days.

SMTA was the only compound whose scalped levels increased over time. After 6 h, there were higher levels compared to 1 h in the C1 samples. C2 had an increase only after 3 days and then remained significantly the same at 7 days. For C3 samples, this increase began after 3 days and then remained stable at 7 days. Finally, the SMTA concentrations were similar at 1 h and 6 h, which then increased after 3 days and stayed the same at 7 days.

#### 2.4.2. Comparison of the Scalping Effect between Closures

The same behaviours of the VSC kinetics and scalping were observed in the experiments conducted on both the model and Shiraz wines.

##### Total Amount of VSC

**Figure 4:**
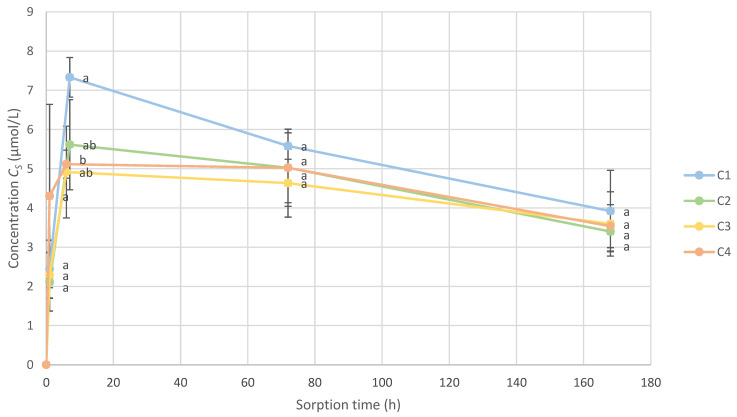
Kinetics of the total amount of VSCs scalped on wine closures ($C_{S}$) in Shiraz wine (the letters a and b represent the significant differences at α = 0.05 between closures at each time of measure).

The scalping effect over time was compared between the four types of closures. At each analysis time, C1, C2, C3, and C4 were compared with one another via an ANOVA statistical analysis using a Student’s *t*-test (Figure 4).

In general, the scalped concentrations of VSCs on the four closures showed no significant differences between them, whether after 1 h of stirring, 3 days, or 7 days. After 6 h of stirring, C1 had a significantly higher $C_{S}$ compared to C4. C2 and C3 displayed no significant differences, with all other closures at 6 h.

##### Comparison of the Scalping Kinetics between Closures for Each VSC

Closures C1, C2, C3 and C4 were compared between each other, for any VSC at any time, but no significant differences were observed on scalped levels.

## 3. Discussion

This experiment showed that several VSCs were able to be scalped at the surface of micro-agglomerated wine closures. While flavour scalping of DMS has already been studied [18], this is the first time that other VSCs such as EtSH, DES, SMTA, ETA, DMDS, and DEDS were reported to be adsorbed at the surface of micro-agglomerated wine closures. These results could represent new insights on the control of the reduction off-flavour formation during storage because a great diversity of VSCs were able to be scalped, with the acetate precursors of EtSH and MeSH, which could prevent their formation during bottle ageing.

### 3.1. Evolution of the Scalping Kinetics of VSC

In both matrices (model and Shiraz wines), flavour scalping of VSCs on micro-agglomerated closures were identified and evaluated, up to 25% of initial DMS levels, specifically by considering whole-surface closures and a scalping duration of 7 days. A previous study had shown similar tendencies [19], with approximately 50% of DMS scalped for natural cork closures after 7 days of soaking. This gap could have been attributed to the difference in the initial concentration of DMS between the two studies, with 267 µg/L in the present experiment and 20 µg/L in [19].

While scalping seemed to increase between 1 h and 6 h, remaining stable afterwards, in the model wine conditions, the analysis of the Shiraz wine showed a decrease after 7 days, probably due to the chemical mechanisms that were not in equilibrium. Indeed, VSCs were likely to interact with other components of the wine, or closures could also interact with other components such as other flavours or polyphenols.

For each VSC, the same behaviour was observed in the model and red wines. Sulphur compounds with very low molecular weights, such as EtSH, DMS, and DES, had decreasing amounts scalped on closures, compared to heavier molecules such as DMDS, SMTA, and ETA, suggesting that molecule volatility could play a role on the ability of flavours to be sorbed. Additionally, previous studies demonstrated that hydrophobicity would be the major effect on flavour scalping, with a higher adsorption observed with more hydrophobic molecules [8]. As the logP for all seven VSCs ranged between 0.7 (SMTA) and 1.8 (DMDS), these molecules could indeed be scalped on micro-agglomerated closures, but these values seemed to be too close to each other to conclude on the role of hydrophobicity on the evolution of each scalped VSC during time.

This experiment was conducted on the whole surface of the closure. However, in bottling conditions, only the mirror (i.e., the part of the cork in contact with the wine during storage) could undergo flavour scalping. Then, levels adsorbed on closures were divided by the factor dividing the surface of the mirror by the surface of the entire closure (factor equal to 9.3) to determine the actual scalping effect during wine evolution (Table 1).

For both the model and red wines, the scalping corresponded to 1% to 5% of the initial VSC concentrations. By extrapolation, the losses accounted for approximately 40% to 80% for the model wine and for 8% to 70% for the Shiraz wine. Therefore, flavour scalping of VSCs could be considered as a minor effect towards the evolution during bottle ageing compared to other phenomena such as VSC chemical reactions or volatility. Consequently, the adsorption of VSCs on micro-agglomerated closures could not significantly impact the formation or disappearance of reduced off-flavour during wine ageing under our conditions.

### 3.2. Comparison of the Scalping Effect between Closures

The aim of this study was to determine if closure permeability, such as the type of closure, was impacting VSC flavour scalping in model and Shiraz wines.

In both matrices, no effect of closure permeability was observed at any time of analysis, except in the Shiraz wine for the sum of VSCs at 6 h of stirring, where C1 had a higher concentration scalped compared to C4. This could mean that the adsorption kinetics could be faster with the tightest closure. Yet, after 3 and 7 days, there were no significant differences between closures, meaning that the permeability of micro-agglomerated cork closures could not influence the scalping phenomenon of VSCs during wine storage.

Other works suggested that flavour scalping could be the cause of a difference in DMS concentration of several Shiraz wines bottled with closures with a high OTR difference [20]. This experiment presented here showed that this hypothesis could probably not explain the differences of DMS levels in these wines.

Surface treatment on closures was also checked for flavour scalping. Indeed, micro-agglomerated closures usually receive a surface treatment to allow the material to adhere to the glass surface. As different kinds of treatments were used on the closures investigated above, the analysis of the same closure processed with different surface treatments (T1, T2, T3, and T4) was performed to check their influence on flavour scalping. In this experiment, four different surface treatments commonly used in the industry were studied, as well as closures with no surface treatments. Closures C1 and C2 were studied in a model wine (Figure S2), and closure C1 was also monitored in a Shiraz wine (Figure S3), after 3 and 7 days of stirring to reach the equilibrium. For the total amount of VSCs, as well as for each of them, surface treatment was not a significant factor for the scalping phenomena, despite the conditions considered.

## 4. Materials and Methods

### 4.1. Chemicals

Ethanol (≥99.8%) was purchased from VWR (Rosny-sous-Bois, France). Tartaric acid (≥99.5%), sodium hydroxide (≥98%), magnesium sulphate heptahydrate (≥99%), and reference standards of ethanethiol (≥98.5%), diethyl sulphide (98%), diethyl disulfide (99%), and thiophene (≥99%) were supplied by Sigma Aldrich (Saint-Quentin-Fallavier, France). For other reference standards, dimethyl sulphide (>99%) and dimethyl disulfide (≥98%) were purchased from Fluka (Charlotte, NC, USA), *S*-methyl thioacetate (≥98%) from Alfa Aesar (Kandel, Germany), and *S*-ethylthioate (97%) from Lancaster Synthesis (Morecambe, UK).

### 4.2. Wine Samples

A Shiraz wine was purchased from a local winery in Côtes-du-Rhône (vintage 2020).

### 4.3. Model Wine

The model wine solution was composed of tartaric acid (5 g), absolute ethanol (120 mL), and milliQ water for 1 L final volume. pH was adjusted to 3.5 using an NaOH solution.

### 4.4. Wine Closures

Four types of micro-agglomerated cork wine closures (Table 2) were provided by DIAM Bouchage (Céret, France). Closure 1 (C1) and closure 2 (C2) had low and medium oxygen transfer rates, respectively (0.19 ± 0.02 mg O_2_/year for closure 1 and 1.15 ± 0.40 mg O_2_/year for closure 2). Closure 3 (C3) and closure 4 (C4) had medium and high oxygen transfer rates (1.07 ± 0.29 mg O_2_/year for closure 3 and 1.79 ± 0.36 mg O_2_/year for closure 4). Oxygen transfer rates were measured by DIAM Bouchage company.

### 4.5. General Workflow for Scalping Study

#### 4.5.1. Development and Optimization of Sample Preparation for VSC Desorption and Subsequent Analysis by GC-MS/MS

Micro-agglomerated closures from Shiraz wines aged for 1 year in bottles were used for this test. Each closure was inserted into a 50 mL Falcon^®^ tube, to which model wine (20 mL) was added. The Falcon tubes were then placed on an agitator and stirred for 1 h at 300 rpm, then 10 mL of the sample was collected into an SPME vial (20 mL). Each Falcon tube containing the closure was then emptied of its liquid, and 20 mL of model wine was added. The same procedure was used at 2 h, 3 h, 6 h, and 18 h.

After agitation, MgSO_4_·7H_2_O (2.5 g) was added to an SPME vial, as described by [21]. In total, 10 mL of the model wine contained in the tube sample was poured into the vial, then an internal standard solution (thiophene; 75 µg/L in absolute ethanol; 80 µL) was added. The vial was then sealed with a Teflon-faced septum and stirred by a vortex.

All SPME vials were then put on GC-MS/MS for analysis.

#### 4.5.2. Artefact Checking from New Closures

**Figure 5:**
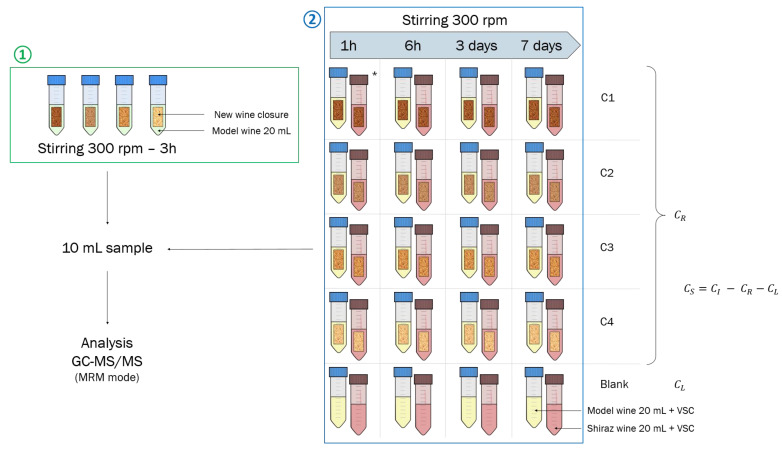
General workflow for the analysis of the closure capacity to adsorb VSC: ① artefact checking; ② trials on model and Shiraz wine matrices ($C_{R}$: concentration remaining in the vial; $C_{I}$: initial concentration; $C_{L}$: concentration lost during stirring; $C_{S}$: concentration scalped on closures). *: one Falcon tube represented corresponds to one sample made in triplicate.

Each type of completely new closure (unused on bottle) was inserted into a 50 mL Falcon tube. Model wine (20 mL) was added to each tube. The tubes were then placed on an agitator and stirred at 300 rpm for 3 h (Figure 5). For each sample, 10 mL was withdrawn and added to a 20 mL SPME vial and prepared as in Section 4.5.1. All trials were conducted in triplicate.

#### 4.5.3. Analysis of Closure Capacity to Sorb VSC under Model and Red Wine Matrices

For each closure, the sorption experiments were made in 2 different matrices: a model wine and a Shiraz wine, both spiked with the same concentrations of VSC (Table 3).

The experiment was performed as follows (Figure 5): each closure was immersed either in model or Shiraz wines (20 mL) spiked with VSC (Table 3), stirred at 300 rpm for t0, 1 h, 6 h, 3 days, and 7 days, and then analysed by GC-MS/MS. Model wine blanks and Shiraz wine blanks were also prepared, with Falcon tubes filled with 20 mL of spiked model wine or spiked Shiraz wine, with no closure. Each modality (duration of stirring) was performed in triplicate.

#### 4.5.4. Analysis by SPME-GC-MS/MS

The following VSCs were analysed by SPME-GC-MS/MS technique: EtSH, DMS, DES, SMTA, DMDS, ETA, and DEDS. The extraction and GC method was developed by [20], whose parameters are briefly given below:

The sample was stirred at 500 rpm for 5 min at 35 °C for homogenization. A 50/30 µm DVB/CAR/PDMS Stableflex 2 cm SPME fibre (Supelco, Bellefonte, PA, USA) was used for extraction. The SPME fibre was inserted into the vial headspace for a 30 min equilibration and was then removed and inserted into the GC injector for 5 min at 240 °C in splitless mode. Chromatographic analysis was performed with a Thermo Trace GC Ultra gas chromatograph coupled with a TSQ 8000 triple quadrupole mass spectrometer (Thermo Scientific, Waltham, MA, USA) equipped with a 30 m × 0.25 mm I.D × 1.00 µm film thickness ZB-WAX fused-silica capillary column (Phenomenex, Le Pecq, France), with a constant helium flow of 0.5 mL/min. The oven temperature program was as follows: 35 °C held for 5 min, heated to 40 °C at a rate of 1 °C/min, held for 1 min, then heated to 240 °C at a rate of 10 °C/min, with a final hold time of 1 min.

The mass spectrometer was equipped with an electron impact ionization source (EI). MS acquisition was performed in multiple reaction monitoring (MRM) mode. The optimized transitions and collision energies are reported in [20]. The transfer line and source temperatures were both set at 220 °C.

## 5. Conclusions

When immerged into model or Shiraz wines spiked with VSCs, micro-agglomerated closures were confirmed to scalp these volatiles at their surfaces. In general, the tendencies to sorb VSCs increased over time, with a higher sorption rate between 1 h and 6 h. Two types of behaviours were found. For EtSH, DMS, DES, and DEDS, after an increase in concentrations at the beginning, levels had a tendency to decrease after 7 days of stirring, suggesting that the polar compounds were unlikely to be adsorbed onto cork. For compounds with higher molecular weights, such as SMTA, ETA, and DMDS, the scalping phenomena remained stable after 6 h up to 7 days and even increased after 6 h for SMTA, confirming that flavour compounds volatility could play a role in the scalping phenomenon.

Despite the VSC considered, no significant effect of the permeability of micro-agglomerated closures was established at any time of the experiment.

From a technical point of view, when sorbed VSC amounts were recalculated correspondingly to the closure surface normally in contact with the wine, we determined that the flavour scalping constituted only 2% to 5% of the becoming of VSCs during bottle ageing, making this phenomenon negligible compared to other potential evolutions.

## Figures and Tables

**Table 1:** Extrapolation of the scalping phenomenon of VSCs when put at the mirror scale, compared to initial VSC concentration ($C_{I}$), in model and Shiraz wines.

	Model Wine	Shiraz Wine
$C_{I}$ (µmol/L)	1.56 × 10^1^	1.67 × 10^1^
C1_1h	1%	2%
C1_6h	3%	5%
C1_3d	3%	4%
C1_7d	2%	3%
C2_1h	1%	1%
C2_6h	3%	4%
C2_3d	3%	3%
C2_7d	3%	2%
C3_1h	1%	2%
C3_6h	3%	3%
C3_3d	3%	3%
C3_7d	3%	2%
C4_1h	2%	3%
C4_6h	3%	3%
C4_3d	3%	3%
C4_7d	3%	2%

**Table 2:** Permeability parameters for micro-agglomerated cork closures (OIR: oxygen initial rate; OTR: oxygen transfer rate).

Closure	Closure 1	Closure 2	Closure 3	Closure 4
OIR (mg O_2_)	0.91 ± 0.04	1.98 ± 0.32	1.92 ± 0.21	2.31 ± 0.20
OTR (mg O_2_/year)	0.19 ± 0.02	1.15 ± 0.40	1.07 ± 0.29	1.79 ± 0.36

**Table 3:** List of volatile sulphur compounds added to model and Shiraz wines with their corresponding theorical concentrations (µmol/L).

Compounds	Abbreviations	Theorical Concentrations (µmol/L)
Ethanethiol	EtSH	2.4
Dimethyl sulphide	DMS	4.3
Diethyl sulphide	DES	0.8
S-methylthioacetate	SMTA	3.4
Dimethyldisulfide	DMDS	0.9
S-ethylthioacetate	ETA	0.7
Diethyldisulfide	DEDS	0.6

## Data Availability

Not applicable.

## Supplementary Materials

The following supporting information can be downloaded at: https://www.mdpi.com/article/10.3390/molecules28135094/s1, Figure S1: Evolution of total VSC desorption in model wine depending on the desorption time (1 h, 2 h, 3 h, 6 h, and 18 h) and the type of closure. Each analysis was performed in monoplicate (the letters a, b, and c represent the significant differences at α = 0.05 between each time of desorption for the totality of closures); Figure S2: Evolution of total VSC in model wine and extrapolations for C1 closure (A) and C2 closure (B), depending on the stirring time and surface treatments (T0: no surface treatment; T1 to T4: closures with surface treatments; the letters a and b represent the significant differences at α = 0.05 between stirring times for each surface treatment and the blanks); Figure S3: Evolution of total VSC in Shiraz wine and extrapolations for C1 closure, depending on the stirring time and surface treatments (T0: no surface treatment; T1 to T4: closures with surface treatments; the letters a and b represent the significant differences at α = 0.05 between stirring times for each surface treatment and the blanks); Table S1: Mean concentration and standard deviation of volatile sulphur compounds in model and Shiraz wines according to the type of closure and the time of stirring (EtSH: ethanethiol; DMS: dimethyl sulphide; DES: diethyl sulphide; SMTA: *S*-methylthioacetate; DMDS: dimethyl disulfide; ETA: *S*-ethylthioacetate; DEDS: diethyl disulfide).
