# Supplementary material for: New Insights on the Scalping Phenomenon of Volatile Sulphur Compounds on Micro-Agglomerated Wine Closures
Source: Molecules. 2023 Jun 29;28(13):5094. doi: 10.3390/molecules28135094 (PMC10343764; doi:10.3390/molecules28135094)
Supplement: Supplementary file 1 [file molecules-28-05094-s001.zip › molecules-2465092-supplementary.pdf]

molecules

Article

New Insights on the Scalping Phenomenon of Volatile Sulphur Compounds on Micro-Agglomerated Wine Closures

Rémi De La Burgade [1], Valérie Nolleau [1], Teddy Godet [1], Nicolas Galy [2], Dimitri Tixador [2], Christophe Loisel [2], Nicolas Sommerer [1] and Aurélie Roland [1,*]

[1] SPO, Univ Montpellier, INRAE, Institut Agro, 34060 Montpellier, France; remi.de-la-burgade@supagro.fr (R.D.L.B.); valerie.nolleau@inrae.fr (V.N.); teddy.godet@inrae.fr (T.G.); nicolas.sommerer@inrae.fr (N.S.)
[2] DIAM Bouchage, 3 Rue des Salines, 66400 Céret, France; galy@diam-bouchage.com (N.G.); tixador@diam-bouchage.com (D.T.); loisel@diam-bouchage.com (C.L.)
* Correspondence: aurelie.roland@supagro.fr; Tel.: +33-4-99-61-22-98

Supplementary Information

As a reminder for the Supplementary information, the total amount of Volatile Sulfur Compounds (VSC) refers to the sum of each VSC analysed in this study: ethanethiol (EtSH), dimethyl sulfide (DMS), diethyl sulfide (DES), *S*-methylthioacetate (SMTA), dimethyl disulfide (DMDS), *S*-ethylthioacetate (ETA) and diethyl disulfide (DEDS).

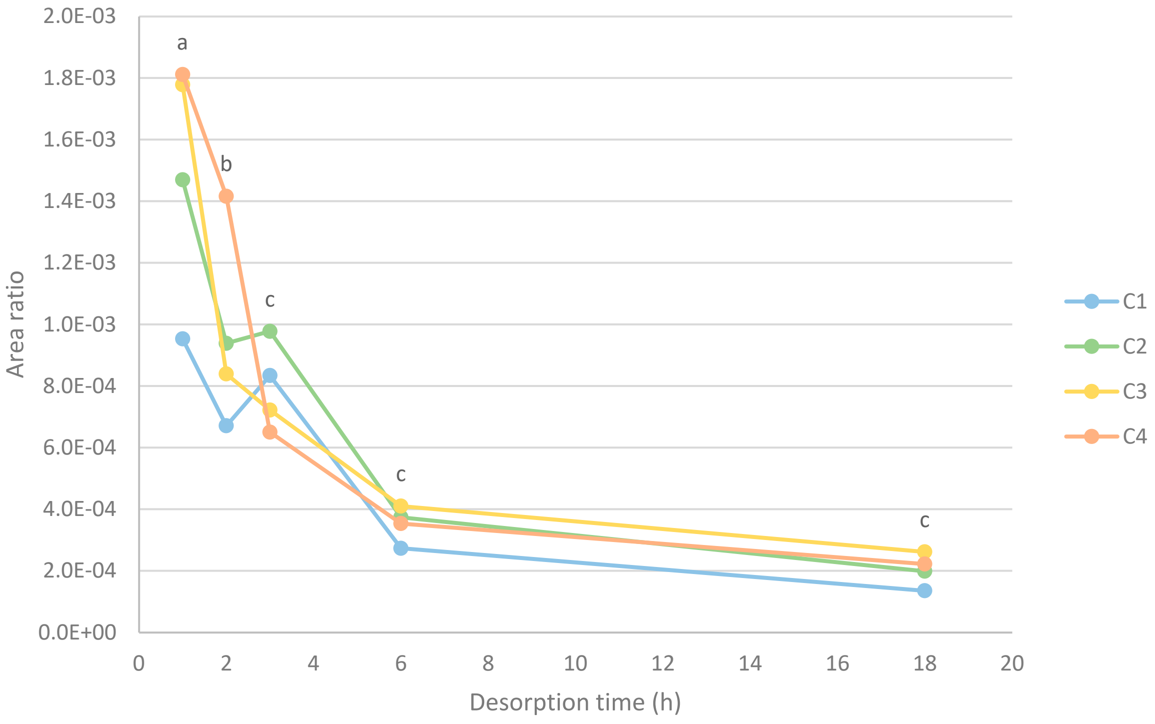

Figure S1. Evolution of total VSC desorption in model wine depending on the desorption time (1h, 2h, 3h, 6h and 18h) and the type of closure. Each analysis was performed in monoplicates (the letters a, b and c represent the significant differences at $\alpha = 0.05$ between each time of desorption for all closures).

Table S1. Mean concentration and standard deviation of volatile sulfur compounds in model and Shiraz wines according to the type of closure and duration of stirring (EtSH: ethanethiol; DMS: dimethyl sulfide; DES: diethyl sulfide; SMTA: *S*-methylthioacetate; DMDS: dimethyl disulfide; ETA: *S*-ethylthioacetate; DEDS: diethyl disulfide). (Ci: initial spiked concentrations of VSC ; Blk: blank sample ; C1, C2, C3 and C4 referred to closures 1, 2, 3 and 4).

	EtSH µmol/L	DMS µmol/L	DES µmol/L	SMTA µmol/L	DMDS µmol/L	ETA µmol/L	DEDS µmol/L
				**Model wine**			
**Ci**	2.37 ± 0.01	4.62 ± 0.06	0.94 ± 0.01	5.07 ± 0.08	1.06 ± 0.13	0.79 ± 0.01	0.75 ± 0.01
**Blk_1h**	1.24 ± 0.34	2.63 ± 0.56	0.49 ± 0.03	3.24 ± 0.05	0.63 ± 0.06	0.56 ± 0.22	0.29 ± 0.05
**Blk_6h**	1.76 ± 0.07	3.05 ± 0.13	0.34 ± 0.03	3.28 ± 0.19	0.59 ± 0.01	0.64 ± 0.03	0.15 ± 0.01
**Blk_3d**	1.25 ± 0.14	1.92 ± 0.21	0.03 ± 0.01	2.66 ± 0.30	0.33 ± 0.00	0.55 ± 0.04	0.07 ± 0.00
**Blk_7d**	0.80 ± 0.34	0.94 ± 0.21	0.00 ± 0.00	3.04 ± 0.55	0.24 ± 0.09	0.45 ± 0.09	0.06 ± 0.01
**C1_1h**	1.08 ± 0.15	2.80 ± 0.26	0.36 ± 0.01	3.00 ± 0.08	0.43 ± 0.04	0.40 ± 0.22	0.12 ± 0.02
**C1_6h**	0.69 ± 0.14	1.65 ± 0.21	0.12 ± 0.00	2.52 ± 0.09	0.25 ± 0.01	0.38 ± 0.14	0.07 ± 0.01
**C1_3d**	0.06 ± 0.01	0.06 ± 0.01	0.00 ± 0.00	1.80 ± 0.03	0.09 ± 0.00	0.26 ± 0.01	0.04 ± 0.00
**C1_7d**	0.02 ± 0.00	0.01 ± 0.00	nd	2.46 ± 0.15	0.05 ± 0.01	0.15 ± 0.07	0.04 ± 0.00
**C2_1h**	1.19 ± 0.09	2.88 ± 0.23	0.35 ± 0.02	2.96 ± 0.09	0.44 ± 0.03	0.55 ± 0.02	0.10 ± 0.00
**C2_6h**	0.95 ± 0.07	1.76 ± 0.11	0.11 ± 0.01	2.38 ± 0.23	0.25 ± 0.02	0.37 ± 0.16	0.07 ± 0.00
**C2_3d**	0.14 ± 0.02	0.09 ± 0.02	nd	1.86 ± 0.01	0.09 ± 0.01	0.26 ± 0.01	0.04 ± 0.00
**C2_7d**	0.02 ± 0.00	0.01 ± 0.00	nd	1.83 ± 0.06	0.06 ± 0.01	0.17 ± 0.07	0.04 ± 0.00
**C3_1h**	0.82 ± 0.03	2.66 ± 0.17	0.35 ± 0.02	3.46 ± 0.36	0.44 ± 0.03	0.55 ± 0.01	0.12 ± 0.01
**C3_6h**	0.64 ± 0.11	1.58 ± 0.23	0.11 ± 0.00	2.84 ± 0.05	0.29 ± 0.04	0.39 ± 0.17	0.08 ± 0.01
**C3_3d**	0.10 ± 0.01	0.08 ± 0.01	0.00 ± 0.00	1.82 ± 0.02	0.11 ± 0.00	0.26 ± 0.01	0.04 ± 0.00
**C3_7d**	0.02 ± 0.00	0.01 ± 0.00	nd	1.66 ± 0.07	0.06 ± 0.01	0.15 ± 0.07	0.04 ± 0.00
**C4_1h**	0.75 ± 0.15	2.16 ± 0.50	0.33 ± 0.02	3.05 ± 0.13	0.39 ± 0.04	0.35 ± 0.17	0.13 ± 0.02
**C4_6h**	0.68 ± 0.04	1.57 ± 0.03	0.10 ± 0.00	2.59 ± 0.09	0.27 ± 0.01	0.44 ± 0.01	0.07 ± 0.00
**C4_3d**	0.06 ± 0.01	0.05 ± 0.00	0.00 ± 0.00	1.57 ± 0.34	0.09 ± 0.00	0.23 ± 0.01	0.04 ± 0.00
**C4_7d**	0.01 ± 0.00	0.01 ± 0.00	nd	1.45 ± 0.07	0.04 ± 0.00	0.10 ± 0.04	0.04 ± 0.00
				**Shiraz wine**			
**Ci**	1.23 ± 0.29	5.90 ± 0.96	0.89 ± 0.08	5.42 ± 1.33	0.94 ± 0.17	0.85 ± 0.01	1.08 ± 0.02

**Blk_1h**	1.00	±	0.06	5.90	±	0.22	0.74	±	0.03	6.14	±	0.13	0.76	±	0.21	0.75	±	0.31	0.72	±	0.05
**Blk_6h**	0.40	±	0.05	5.40	±	0.35	0.45	±	0.00	6.30	±	0.37	0.67	±	0.02	1.02	±	0.19	0.49	±	0.06
**Blk_3d**	0.85	±	0.04	1.85	±	0.17	0.01	±	0.01	5.74	±	0.13	0.29	±	0.24	0.71	±	0.13	0.18	±	0.02
**Blk_7d**	0.18	±	0.03	0.59	±	0.03	nd			5.12	±	0.30	0.24	±	0.10	0.30	±	0.24	0.11	±	0.01
**C1_1h**	0.79	±	0.08	4.76	±	0.25	0.56	±	0.02	5.53	±	0.13	0.41	±	0.02	0.80	±	0.07	0.32	±	0.01
**C1_6h**	0.05	±	0.01	2.44	±	0.04	0.17	±	0.00	4.18	±	0.46	0.21	±	0.01	0.13	±	0.12	0.15	±	0.01
**C1_3d**	0.20	±	0.16	0.28	±	0.34	0.01	±	0.01	2.79	±	0.12	0.08	±	0.05	0.21	±	0.01	0.06	±	0.00
**C1_7d**	0.02	±	0.00	0.05	±	0.00	nd			2.19	±	0.36	0.08	±	0.01	0.18	±	0.06	0.05	±	0.00
**C2_1h**	0.77	±	0.07	5.03	±	0.30	0.58	±	0.04	5.54	±	0.35	0.47	±	0.00	0.77	±	0.05	0.34	±	0.03
**C2_6h**	0.08	±	0.01	3.14	±	0.05	0.19	±	0.00	4.69	±	0.08	0.29	±	0.01	0.30	±	0.06	0.19	±	0.01
**C2_3d**	0.47	±	0.02	0.32	±	0.33	0.01	±	0.01	3.16	±	0.22	0.07	±	0.14	0.34	±	0.28	0.06	±	0.00
**C2_7d**	0.02	±	0.01	0.06	±	0.01	nd			2.62	±	0.12	0.07	±	0.02	0.25	±	0.05	0.05	±	0.00
**C3_1h**	0.91	±	0.16	4.76	±	0.30	0.56	±	0.01	5.52	±	0.05	0.47	±	0.17	0.77	±	0.01	0.30	±	0.03
**C3_6h**	0.07	±	0.01	3.46	±	0.06	0.28	±	0.00	4.87	±	0.07	0.25	±	0.00	0.47	±	0.10	0.17	±	0.01
**C3_3d**	0.45	±	0.02	0.58	±	0.18	0.01	±	0.02	3.35	±	0.25	0.08	±	0.06	0.26	±	0.29	0.07	±	0.00
**C3_7d**	0.02	±	0.01	0.10	±	0.01	nd			2.56	±	0.12	0.05	±	0.01	0.11	±	0.10	0.05	±	0.00
**C4_1h**	0.71	±	0.09	3.96	±	0.88	0.45	±	0.04	5.10	±	0.40	0.38	±	0.04	0.38	±	0.30	0.35	±	0.07
**C4_6h**	0.05	±	0.01	3.29	±	0.05	0.19	±	0.00	4.86	±	0.12	0.31	±	0.00	0.52	±	0.04	0.16	±	0.02
**C4_3d**	0.41	±	0.02	0.27	±	0.13	0.01	±	0.02	3.25	±	0.04	0.08	±	0.09	0.32	±	0.13	0.06	±	0.00
**C4_7d**	0.01	±	0.01	0.05	±	0.01	nd			2.60	±	0.08	0.07	±	0.02	0.16	±	0.09	0.05	±	0.00

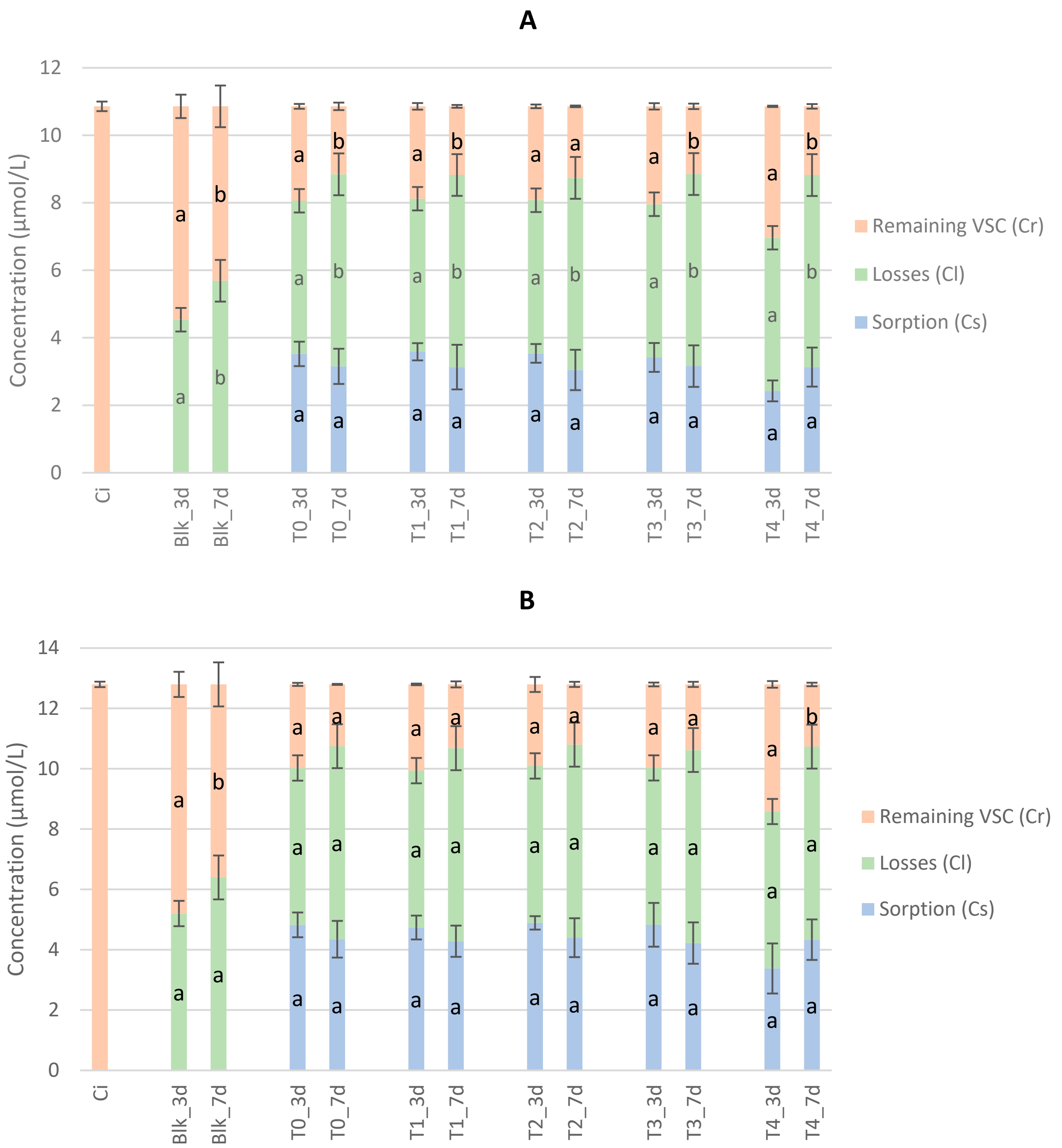

Figure S2. Evolution of total VSC in model wine and extrapolations for C1 closure (A) and C2 closure (B), depending on stirring duration and surface treatments (T0: no surface treatment; T1 to T4: closures with surface treatments ; the letters a and b represent the significant differences at $\alpha = 0.05$ between stirring durations for each surface treatment and the blanks).

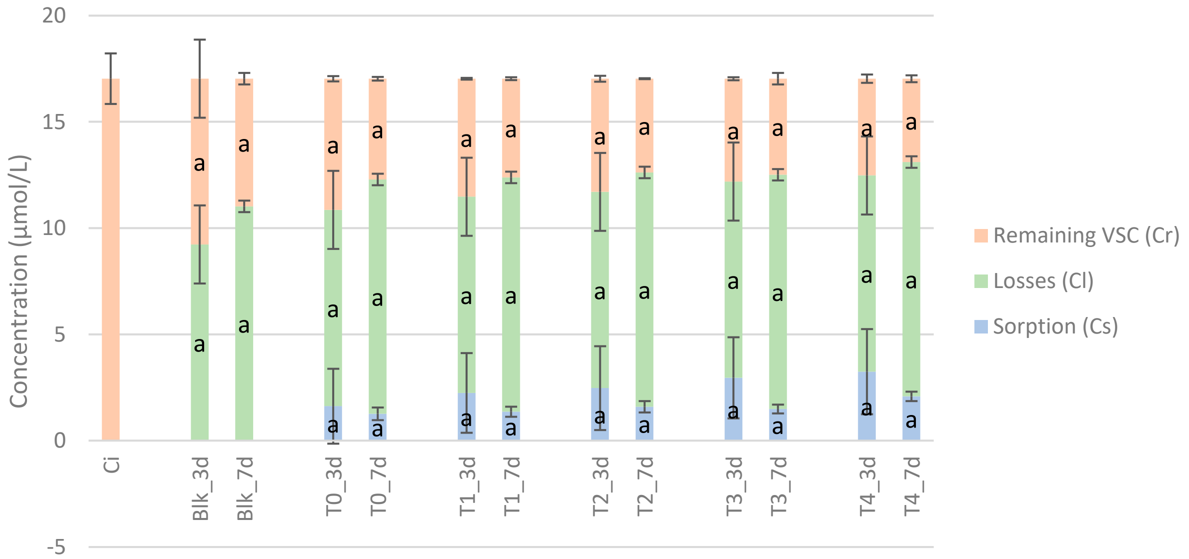

Figure S3. Evolution of total VSC in Shiraz wine and extrapolations for C1 closure, depending on stirring duration and surface treatments (T0: no surface treatment; T1 to T4: closures with surface treatments ; the letters a and b represent the significant differences at $\alpha = 0.05$ between stirring durations for each surface treatment and the blanks).
